# Retrograde Migration of an Au-198 Grain to the Submandibular Gland Post Brachytherapy Treatment of Floor of Mouth Cancer

**DOI:** 10.7759/cureus.31904

**Published:** 2022-11-26

**Authors:** Hitomi Nojima, Atsushi Kaida, Takeshi Kuroshima, Ryo-ichi Yoshimura, Masahiko Miura

**Affiliations:** 1 Department of Dental Radiology and Radiation Oncology, Graduate School of Medical and Dental Sciences, Tokyo Medical and Dental University, Tokyo, JPN; 2 Department of Oral and Maxillofacial Surgical Oncology, Graduate School of Medical and Dental Sciences, Tokyo Medical and Dental University, Tokyo, JPN; 3 Department of Radiation Therapeutics and Oncology, Graduate School of Medical and Dental Sciences, Tokyo Medical and Dental University, Tokyo, JPN

**Keywords:** floor of mouth cancer, retrograde migration, submandibular gland, wharton’s duct, brachytherapy, au-198 grain

## Abstract

At our institution, radiation oncologists routinely treat early-stage oral cancer with low-dose-rate brachytherapy (LDR-BRT) using Au-198 grains. In this report, we show a unique case of a patient with a gold grain located within the submandibular gland, found incidentally during follow-up after LDR-BRT for floor of mouth cancer. One month after the implant, he showed sialadenitis-like symptoms, but the pain resolved two months later. All the grains were detected around the anterior sublingual area by computed tomography (CT) four months after the implant. Unexpectedly, 11 months after the implant, CT revealed that a grain was located in an intraglandular site of the submandibular gland. This finding clearly demonstrates that the grain entered Wharton’s duct and retrogradely migrated to the submandibular gland through the duct. As a mechanism of the calculus formation within Wharton’s duct, retrograde migration of foreign bodies to the inside of the duct has been proposed. Our incidental finding after LDR-BRT highlights the need for monitoring post-LDR-BRT using Au-198 grains for the treatment of floor of mouth cancer and sheds additional light on retrograde theory within Wharton’s duct.

## Introduction

Brachytherapy (BRT) is performed for early-stage oral cancer as a definitive radiotherapy. In addition to being equivalent to the local control achieved by surgery, it retains oral function, resulting in a high quality of life with minimal side effects [1-4]. This is accomplished through the ideal distribution of a high dose of radiation to tumor tissues and a low dose to surrounding tissues, which is not achieved by external irradiation. Two types of BRT are primarily used to treat oral cancer: low dose rate (LDR) (0.4-2 Gy/h) and high dose rate (12 Gy/h) [5]. The former is a well-established approach, and radioisotopes like Au-198 grains or low-activity Ir-192 hairpins are directly implanted in oral tumor tissues. Au-198 grains are permanently implanted, but Ir-192 hairpins are removed after approximately five days. Thus, tumors are continuously irradiated at an LDR. In the latter case, catheters or flexible tubes are implanted, and tumors are intermittently irradiated twice daily by a remote afterloading system using high-activity Ir-192. This sequence is usually repeated for five days. Although this approach has some advantages in terms of radiation exposure to operators and management of hospitalized patients, we have been using the low-dose-rate brachytherapy (LDR-BRT) method, particularly because the implant of the Au-198 grain is a minimally invasive procedure completed in 30 minutes under local anesthesia. Thus, it is especially useful for elderly patients [6,7]. Here, we report a rare case in which a gold grain moved to the submandibular gland during follow-up after LDR-BRT treatment of floor of mouth cancer using Au-198 grains.

## Case presentation

**Figure 1:**
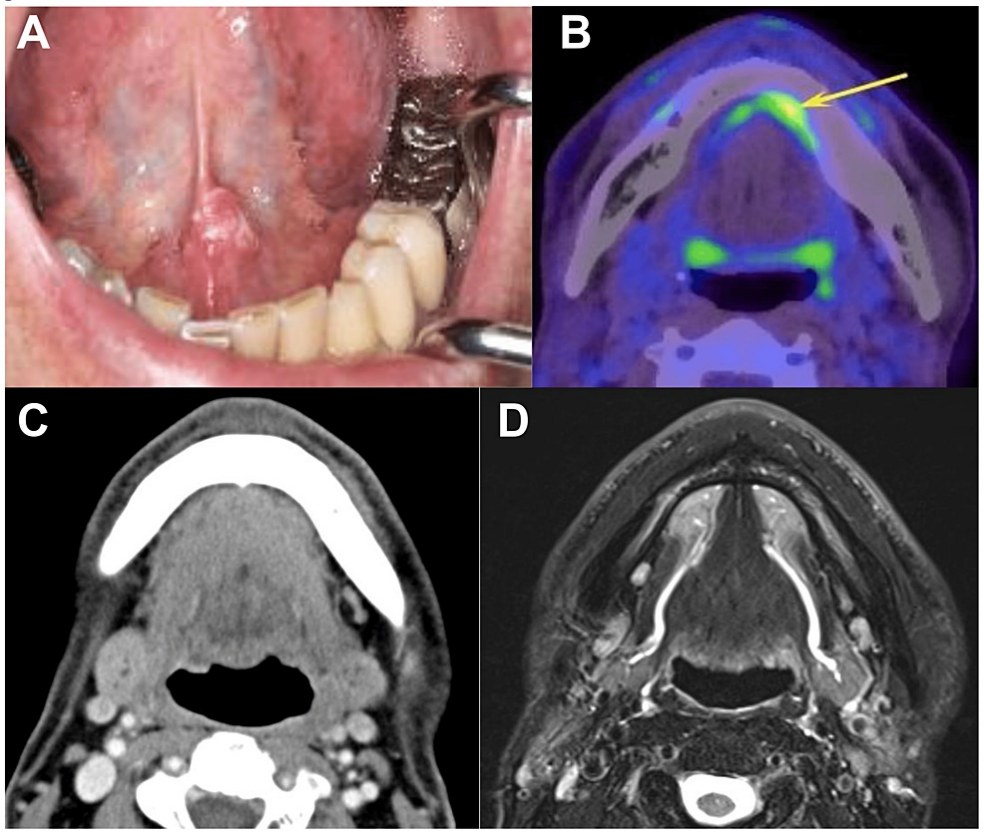
Pretreatment findings. Intraoral photograph: a mass in the left floor of the mouth (A). PET/CT: uptake on the left of the floor of the mouth (yellow arrow) (B). Contrast-enhanced CT: no abnormalities in the left submandibular gland (C). T2WI(FS) MRI: drain from the submandibular glands into the oral cavity via Wharton’s duct (D). PET/CT: Positron emission tomography/computed tomography; CT: computed tomography; T2WI(FS): T2-weighted fat-suppressed; MRI: magnetic resonance imaging

A 63-year-old Japanese man noticed a mass on the left side of the floor of his mouth in November 2020. Past medical history was unremarkable. The patient visited the oral surgeon at our hospital and was diagnosed by biopsy with squamous cell carcinoma (SCC). He was referred to our department for brachytherapy in December 2020. Clinical examination revealed a 1.2 × 0.7 cm exophytic mass in the left anterior floor of the mouth (Figure 1A). On palpation, the lesion was firm and not tender. Salivary outflow from the left sublingual caruncle was normal, and no lymph nodes were palpable in the cervical region. 

Panoramic X-ray, contrast-enhanced computed tomography (CT), and contrast-enhanced magnetic resonance imaging (MRI) could not detect the tumor. Fluorine-18-fluorodeoxyglucose positron emission tomography/computed tomography (^18^F-FDG PET/CT) revealed uptake in the left floor of the mouth (SUVmax=5.6) (Figure 1B). No evidence of metastasis was detected at the cervical lymph node, lung, or elsewhere. No abnormalities in the left submandibular gland were observed (Figure 1C), and drainage from the submandibular glands into the oral cavity via Wharton’s duct was clearly detected (Figure 1D). The patient was clinically diagnosed with early stage (T1N0M0) floor of mouth SCC.

**Figure 2:**
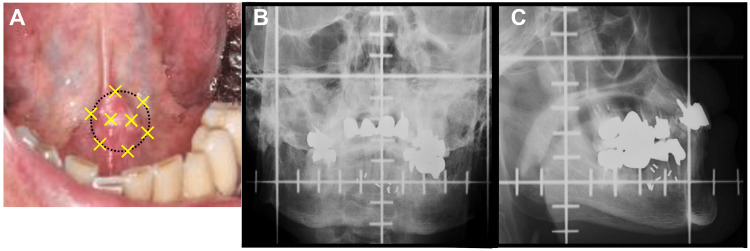
Implant information. Treatment target area (black dotted circle) and distribution of Au-198 grains (yellow cross) (A). Actual distribution of Au-198 grains in posteroanterior (B) and lateral (C) X-ray photographs.

After S-1 (tegafur/gimeracil/oteracil) oral chemotherapy (Σ3, 120 mg, 120 mg/day), in February 2021, eight Au-198 grains were implanted into the left side of the floor of the mouth under local anesthesia, and the patient was hospitalized in isolation for four days. The number of grains was determined as follows. The treatment target area was set as a circle of 2 cm in diameter (Figure 2A), and the radioactivity applied to the area using Ra-226 was first determined according to the Paterson-Parker table [8]. This was then converted to Au-198, resulting in 4.9 mCi/ 10 Gy. At our institution, the total absorption dose of Au-198 grains is usually set to be 80-90 Gy/∞. In order to fall within this range, the number of grains was determined to be eight (83 Gy/∞), considering that the radioactivity per Au-198 grain was 188 MBq at the time of implantation. The depth of the implanted grains was approximately 3 mm from the surface of the mucosa as shown in X-ray photographs in Figure 2B (posteroanterior) and Figure 2C (lateral).

**Figure 3:**
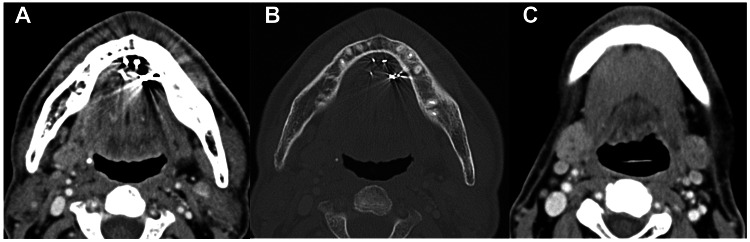
Contrast-enhanced CT four months after the implant. Detection of gold grains in the left floor of the mouth in soft tissue (A) and bone (B) window images. Soft tissue window image at the submandibular gland level (C). CT: Computed tomography

After the LDR-BRT, we followed the patient in an outpatient setting. In March 2021, while apparent mucositis due to acute toxicity had almost resolved, he had recurrent pain in the left distal floor of his mouth after drinking water, and it resolved after 20 minutes. Saliva from the orifice exhibited good flow, but the sublingual fold was hard on palpation. In May 2021, while the pain had disappeared, salivary outflow from the left sublingual caruncle was not clear. In June 2021, four months after the implantation, CT showed no evidence of recurrence in the floor of the mouth and no cervical lymph node metastasis. Implanted gold grains were detected in the left floor of the mouth (Figure 3A, soft tissue window image; Figure 3B, bone window image), and no abnormal findings were noted in the left submandibular gland (Figure 3C). The patient was aware of a slightly dry mouth, and the sublingual fold was hard without pain.

**Figure 4:**
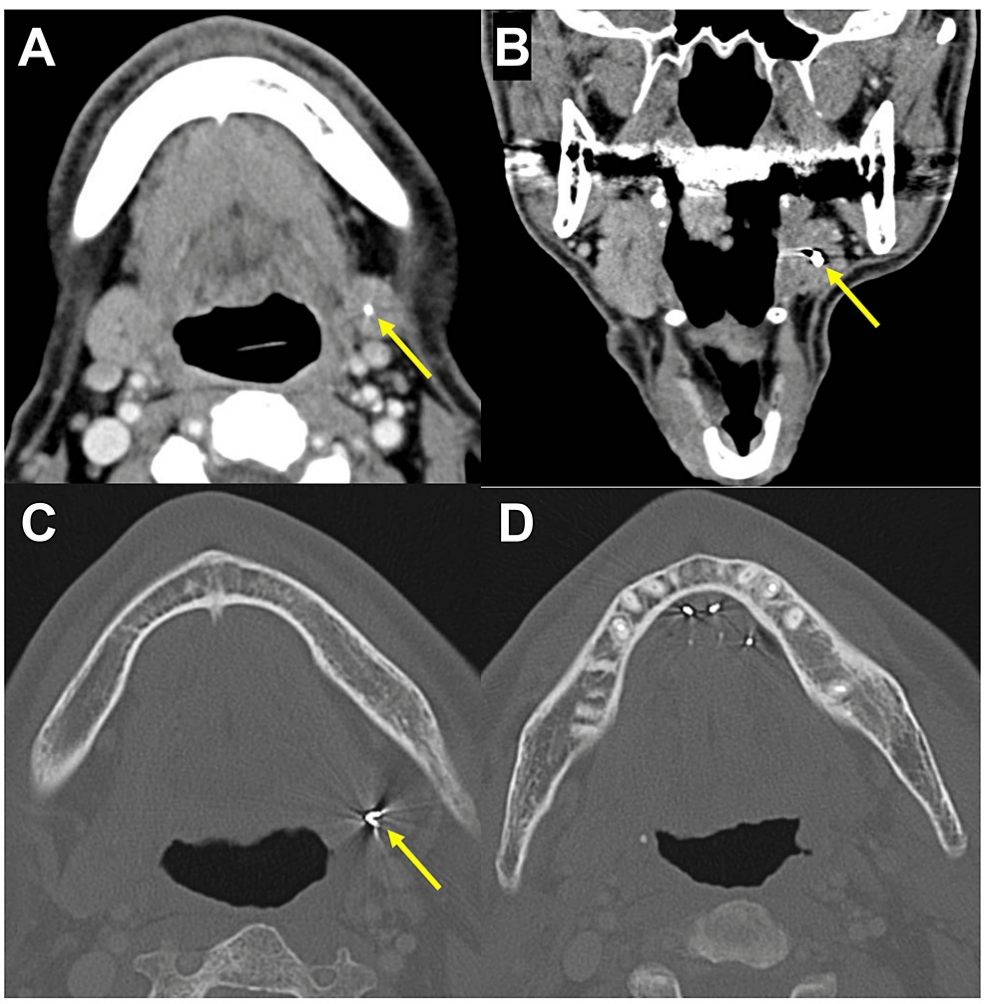
Contrast-enhanced CT 11 months after the implant. Detection of a gold grain in the left submandibular gland in the soft tissue (A, axial; B, coronal) and bone (C, axial) window images (yellow arrows, a gold grain in the submandibular gland). Detection of multiple gold grains in the anterior floor of the mouth in the bone window image (D). CT: Computed tomography

In January 2022, after a careful follow-up, CT showed a sialolith-like structure in the left submandibular gland (Figure 4A, axial, soft tissue window image; Figure 4B, coronal, soft tissue window image; Figure 4C, axial, bone window image). Gold grains were detected at the originally implanted area (Figure 4D, axial, bone window image). Surprisingly, the CT value of the structure was 3071 HU, which is equivalent to metals and was the same as values for gold grains observed in the anterior side of the floor of the mouth (Figure 4D). During follow-up, the patient had never experienced an episode of traumatic injury in his oral cavity. This demonstrated that it was not a sialolith, but one of the implanted gold grains. However, apparent subjective symptoms were not detected.

**Figure 5:**
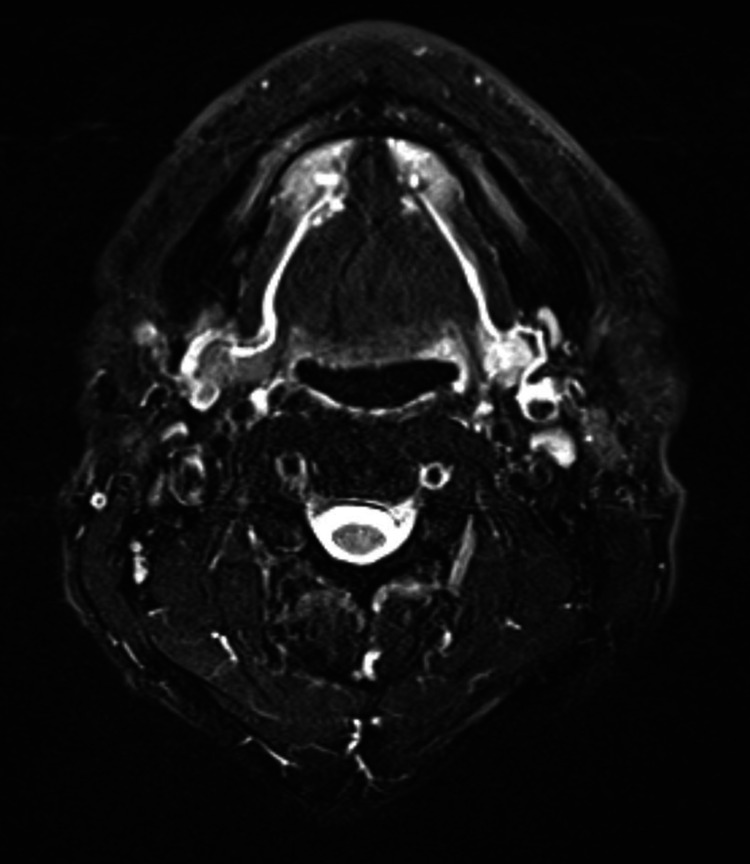
T2WI(FS) MRI one year and four months after the implant. Detection of Wharton’s duct and the submandibular gland. T2WI(FS): T2-weighted fat-suppressed; MRI: magnetic resonance imaging

Five months later, MRI showed saliva throughout Wharton’s duct, but the apparent flow was still unclear near the left sublingual caruncle. In addition, the left submandibular gland showed high signal intensity in the T2-weighted fat-suppressed MRI, suggesting the existence of inflammation (Figure 5).

Except for the short-term pain after drinking water approximately one month after the implant, he had unclear salivary outflow from the left sublingual caruncle and no other significant subjective symptoms. Moreover, he exhibited no radiographic or clinical evidence of cancer recurrence. During our follow-up, he received radiation therapy for primary lung cancer and surgery for gastric cancer, and neither of them recurred.

## Discussion

**Figure 6:**
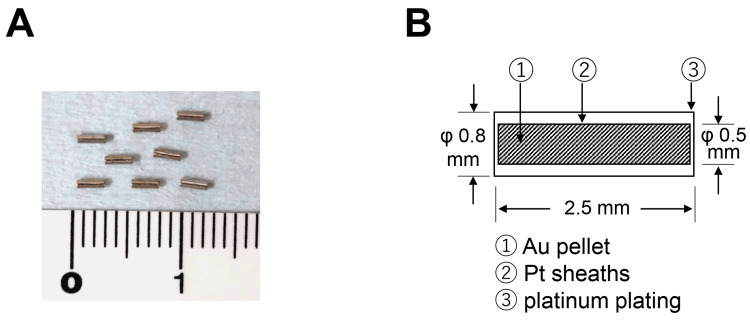
Photograph (A) and schematic presentation (B) of the Au-198 grain

The Au-198 grain consists of an Au pellet and a sheath of Pt coated on platinum plating (Figure 6). In addition to the short half-life of radioactivity of the Au-198 grain (2.7 days), such noble metals are chemically stable and the surface of the grain is quite smooth, which allows permanent implantation to be possible. Although radiation-induced complications such as mucosal ulcer and bone exposure have been reported, there has been no previously reported grain entry into the submandibular gland after a long movement through Wharton’s duct, as in this case [9,10]. We used Au-198 grains with radioactivity of 188 MBq per grain at the time of implantation. It had been present in the floor of the mouth for at least four months and was expected to have decayed below 1.0 × 10^-14^ MBq when it moved to the submandibular gland. Therefore, the direct effect of radioactivity from the Au-198 grain on the submandibular gland can be considered negligible. Fortunately, the patient currently shows no significant subjective symptoms, but we need to carefully follow up to see if submandibular adenitis or duct obstruction worsens in the future.

Gold grains were not detected in the left submandibular gland before BRT (Figure 1A) or four months after BRT (Figure 3C). Notably, CT images 11 months after the implant revealed that a gold grain was located in the submandibular gland. It would be difficult for implanted grains to enter the duct from the orifice on the caruncle. Therefore, it was speculated that the implant needle incidentally pierced the wall of the duct, and a grain was put inside the duct close to the orifice. Analysis of patients with intact salivary gland morphology who underwent digital subtraction sialography revealed an average submandibular duct length of 58 mm and an average diameter of 2.3 mm for the entire duct [11]. Others also reported that the average diameter of the submandibular duct is 1.5-2.7 mm [12-14]. An Au-198 grain is 2.5 mm in length and 0.8mm in diameter. The MRI detected saliva inside the duct when the grain was located within the submandibular gland, which supports the idea that the grain did not completely obturate the duct. The sialadenitis-like symptoms including pain, observed only approximately one month after the implant, might be caused by the originally loose duct clogging as the grain became tight due to the radiation-induced acute inflammation within the duct. After disappearance of the inflammation, the grain could be released from the trap inside the duct. We wondered where the opposite pressure would come from to move it toward the gland against the one moving toward the orifice associated with saliva flow from the gland.

There are some reports that foreign bodies including a fish bone [15], shrimp [16], and blade of grass are found in sialoliths formed inside Wharton’s duct or the submandibular gland [17]. One of the possibilities is that some sialoliths might result from the traumatic migration of foreign bodies into Wharton’s duct or a retrograde migration from the orifice, leading to the submandibular gland on rare occasions. To explain it, a sphincter system around the orifice or a sphincter-like system within the duct must be assumed. Marchal et al. reported that in most cases (90%), a sphincter system was identified approximately 3 cm from the entrance to Wharton’s duct [18]. Su et al. speculated that the variation of the sphincter-like structure within Wharton’s duct may induce a retrograde migration of a fish bone into the submandibular gland against the saliva flow [19]. Supporting this idea, Amano et al. showed that smooth muscle is dispersed throughout the human Wharton’s duct and may play a minor role in regulating the saliva flow [20]. Taken together, our case may have been induced by a stronger pressure toward the gland as a result of an imbalance originating from the sphincter-like system existing in the duct.

## Conclusions

Our case may be the first report of a foreign body traveling in a retrograde fashion almost the full length of Wharton’s duct presumably from the site near the orifice to the center of the submandibular gland. Based on this rare experience, it may be recommended to carefully check the running direction of Wharton’s duct or insert a bougie into Wharton’s duct during the implant to avoid direct implant to the duct. This case report calls attention to using LDR-BRT with Au-198 grains, and also, unexpectedly, the existence of a potential function of Wharton’s duct, which has the ability to generate forces and pressures sufficient to produce retrograde flow toward the submandibular gland. Our new clinical findings may shed additional light on the retrograde theory.
